# Removal of Artistic and Cosmetic Tattoos Using Q‐Switched Nd:YAG Laser

**DOI:** 10.1111/jocd.71038

**Published:** 2026-07-09

**Authors:** Shaked Menashe, Sharon Moscovici, Omer Dor, Yoad Govrin, Gerta Kapxhiu, Arminda Avdulaj

**Affiliations:** ^1^ Assaf Harofeh Medical Center Tel Aviv Israel; ^2^ Eden Laser Clinic Tiranë Albania; ^3^ San Luca Medical Clinic Tiranë Albania

**Keywords:** anatomic location, artistic tattoos, cosmetic tattoos, Q‐switched Nd:YAG laser, tattoo clearance, tattoo removal

## Abstract

**Background:**

Q‐switched 1064/532‐nm Nd:YAG laser therapy for tattoo removal is common but yields variable results across patients and tattoos. Identifying factors related to poor clearance in clinical practice may be more valuable than simply describing overall efficacy.

**Methods:**

A multicenter retrospective analysis was conducted among 111 adult participants who received artistic/cosmetic tattoo removal using a 1064/532‐nm Q‐switched Nd:YAG protocol. Retreatment tattoos were included in the main cohort, while resistant tattoos could be combined with an additional fractional CO_2_ procedure during the same treatment session. The proportion of patients receiving adjunctive fractional CO_2_ rescue treatment was recorded, and a sensitivity analysis was performed after excluding these patients. All participants received 1–8 sessions (average ± SD, 2.5 ± 1.55 sessions). Percentage clearance, assessed by independent blinded physicians using standardized pre‐ and post‐treatment photographs, served as the dependent variable. Associations between clearance and different patient‐, tattoo‐, and treatment‐related parameters were evaluated in both univariable and multivariable linear regression models. Adverse events were analyzed descriptively.

**Results:**

Average photographic clearance was 64.1% ± 18.1%. After controlling for confounders, tattoo localization on the trunk (+16.3%, *p* < 0.001) and face/neck (+16.1%, *p* = 0.002) was associated with higher clearance, while smaller size was observed for tattoos on the trunk, but not face/neck and limbs. Most patients (88.3%) did not experience any adverse events; those that occurred were rare and mostly included inflammatory hyperpigmentation, prolonged erythema, hypopigmentation, and scar formation.

**Conclusion:**

Q‐switched Nd:YAG laser‐centered tattoo removal treatment provided effective photo‐clearance accompanied by minor adverse events. Anatomic location was found to be the factor with the greatest impact on treatment effectiveness, as limb tattoos had lower clearance than those on the trunk and face/neck.

## 1 Introduction

In parallel with the increasing prevalence of tattoos, there is also a rising need for tattoo removal procedures [1, 2, 3]. Moreover, several dermatologic problems may arise, including allergies, infections, pigment dispersion, granuloma formation, and diagnostic challenges during the assessment of skin cancer or regional lymph nodes [2]. Thus, tattoo removal represents an expanding issue in the medical field, both from a clinical point of view and in the scientific literature [3, 4].

Among the various treatment techniques, laser treatment is preferred for its ability to target the tattoo ink while limiting damage to the adjacent skin. The role of Q‐switched devices in tattoo removal therapy stems from their ability to efficiently fragment tattoo ink without causing scarring or changes in skin pigmentation, thanks to their fast nanosecond pulses [5]. It is common knowledge that the clinical application of the dual‐wavelength Q‐switched Nd:YAG laser system allows removal of most artistic tattoos by using 1064 nm to treat the darkest pigments and 532 nm for light‐colored pigments [5, 6].

Although widely used in clinical practice, treatment response is heterogeneous, and complete tattoo removal cannot always be achieved. Factors affecting clinical results, such as tattoo area, ink color, age, density, location, and previous treatments, have been identified, although they do not apply to all cases [7, 8, 9]. Therefore, clinicians and patients prefer to know whether the treatment can effectively remove the tattoo rather than identify which factors may reduce the response.

This is why the current multicenter retrospective cohort study assessed the photographic clearance rate and adverse events of tattoo removal in adult patients undergoing Q‐switched 1064/532‐nm Nd:YAG treatment for their artistic/cosmetic tattoos. Thanks to the inclusion of previously treated tattoos in the cohort and to the additional rescue treatments with fractional CO_2_ laser in resistant tattoos, this study aimed to investigate the real‐world effectiveness of the procedure. More specifically, the objective of the study was to determine which clinical factors affect treatment response, with particular reference to the tattoo location.

## 2 Materials and Methods

### 2.1 Study Design and Setting

This was a multicenter retrospective cohort study of consecutive adult patients treated for tattoo removal at participating centers in Israel and Albania. The study was approved by the Institutional Ethics Committee of Shamir Medical Center (approval no. ASF‐0202‐24), and written informed consent for treatment and clinical photography was obtained from all patients.

### 2.2 Participants

Adults aged 18 years or older who underwent laser treatment to remove artistic or cosmetic tattoos were eligible for inclusion. Both treatment‐naïve and previously treated tattoos were included to reflect routine clinical practice. Exclusion criteria included isotretinoin use and prior gold‐salt exposure within 6 months before treatment because of the known risk of post‐treatment scarring and pigmentary complications [9, 10]. A total of 111 patients were included in the analysis.

### 2.3 Treatment Protocol

Treatment was performed using a Q‐switched Nd:YAG platform (Harmony XL Pro with QSW ClearLift Pro applicator; Alma Lasers, Israel) at 1064 nm and 532 nm, with wavelength selection based on tattoo pigmentation. Black or dark tattoos were treated primarily with 1064 nm, whereas non‐black or multi‐colored tattoos were treated with 532 nm alone or with both wavelengths when clinically indicated.

**FIGURE 1:**
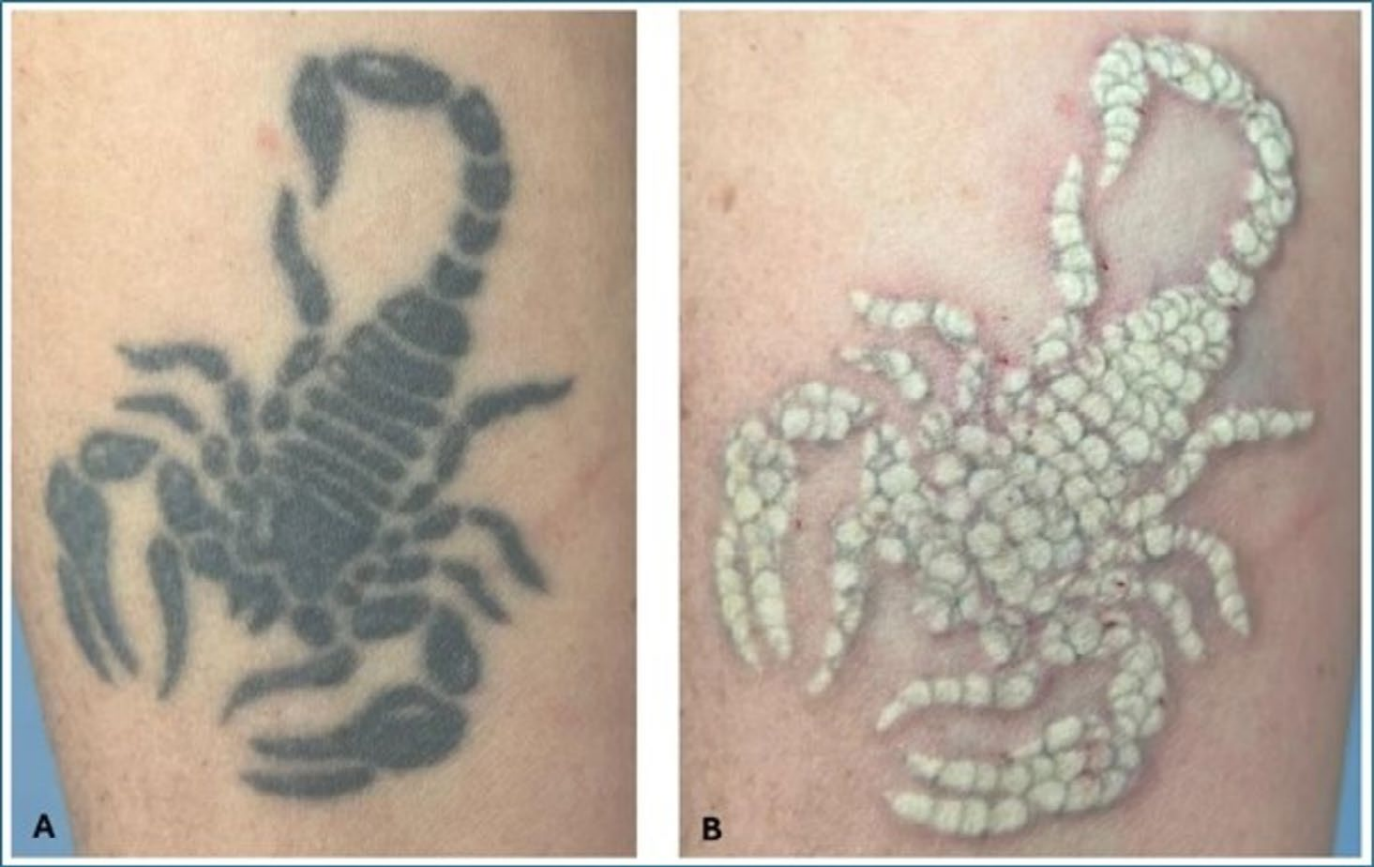
Tattoo (A) before and (B) at the end of the treatment session. “Frosted” appearance of the treated area often indicates adequate energy delivery to the pigment.

For 1064‐nm treatment, the spot size ranged from 2 to 5 mm, the pulse energy from 800 to 1200 mJ/pulse, and the repetition rate from 1 to 3 Hz. For 532‐nm treatment, the spot size ranged from 3 to 4 mm, the pulse energy from 400 to 600 mJ/pulse, and the repetition rate from 1 to 2 Hz. In general, the first treatment session was initiated using the widest spot size and the lowest settings that produced an immediate whitening or frosting response (Figure 1), after which parameters were adjusted according to pigment density and clinical response in subsequent sessions.

The number of treatment sessions ranged from 1 to 8, with a mean of 2.5 ± 1.55. Intervals between sessions varied in routine practice, typically 6–7 weeks for artistic tattoos and 4–5 weeks for eyebrow tattoos, with a minimum recommended interval of 1 month. Tattoos judged to have insufficient response after 3 sessions were classified as resistant. In these cases, adjunctive fractional CO_2_ laser treatment (Alma Pixel CO_2_, Alma Lasers, Israel) could be performed before Nd:YAG/KTP treatment in the same session. Fractional CO_2_ settings ranged from 8 to 16 W with a pulse duration of 1.2–1.6 ms. These rescue cases were retained in the primary analysis to preserve the pragmatic, real‐world nature of the cohort. Adjunctive fractional CO_2_ rescue treatment was performed in 44 of 111 patients (40%). Because fractional CO_2_ treatment may independently influence tattoo clearance, these patients were identified as a separate subgroup for sensitivity analysis.

### 2.4 Periprocedural Care

Cosmetics, makeup, and other surface products were removed before each session to minimize interference with laser‐skin interaction. Local anesthesia was not routinely used but was provided on request in the form of topical lidocaine cream or local injection. After treatment, patients were prescribed a nonadhesive dressing with topical antibiotic and steroid cream for 1 week, and sunscreen use was recommended for 2 months.

### 2.5 Outcome Measures

The primary outcome was percentage tattoo clearance, assessed using standardized before‐and‐after clinical photographs. Before assessment, photographs were de‐identified and coded with random study numbers. Clearance was evaluated independently by two physicians who were not involved in the treatment procedures and were blinded to patient identity, number of treatment sessions, treatment parameters, prior treatment status, fractional CO_2_ rescue‐treatment status, and clinical outcomes. Because the anatomical site was visible in some photographs, complete blinding to body region was not always feasible. Clearance was recorded as a continuous estimate of the reduction in visible pigment. The mean of the two assessors' ratings was used for analysis; discrepancies > 15 percentage points were resolved by consensus or by a third independent reviewer. Tattoo size was measured and categorized as small (1–10 cm^2^), medium (11–50 cm^2^), large (51–100 cm^2^), or XL (> 100 cm^2^). Tattoo color was classified as monochromatic or multi‐colored. For analysis, anatomical locations were grouped into limbs, head/neck, and torso based on the primary tattoo site. Additional recorded variables included age, sex, Fitzpatrick skin type, tattoo age, number of sessions, and, where applicable, the interval between sessions.

Safety outcomes included post‐inflammatory hyperpigmentation, hypopigmentation, prolonged erythema, and scarring, as documented in the medical record and follow‐up photographs.

### 2.6 Statistical Analysis

All statistical analyses were performed using R Studio version 4.3.3. Continuous variables are presented as mean ± standard deviation, and categorical variables as frequencies and percentages. Statistical significance was defined as a two‐sided *p*‐value < 0.05.

Inter‐rater reliability for photographic clearance assessment was evaluated using a two‐way random‐effects, absolute‐agreement, average‐measures intraclass correlation coefficient (ICC[2,k]), with 95% confidence intervals. Tattoo clearance percentage was analyzed as the primary efficacy outcome. Univariable linear regression was first used to explore associations between clearance and candidate predictors. Variables showing significant associations in univariable analyses were then entered into a multivariable linear regression model, which was additionally adjusted for age, skin type, and tattoo size to account for potential confounding. To evaluate the potential confounding effect of adjunctive fractional CO_2_ rescue treatment, a sensitivity analysis was performed by repeating the main multivariable linear regression model after excluding all patients who received fractional CO_2_ treatment. This model used the same covariates as the primary model. The robustness of the primary findings was assessed by comparing the direction, magnitude, and statistical significance of the adjusted body‐region effects between the full cohort and the Nd:YAG‐only cohort. Because interval data were available only for patients who underwent more than one treatment session, a second multivariable model was constructed in that subgroup to assess the possible effect of treatment interval. Where appropriate, pairwise comparisons of estimated marginal means were performed using Tukey‐adjusted multiple comparisons.

Safety outcomes were summarized descriptively. An exploratory one‐way ANOVA was used to examine whether clearance differed across reported side‐effect categories.

## 3 Results

**FIGURE 2:**
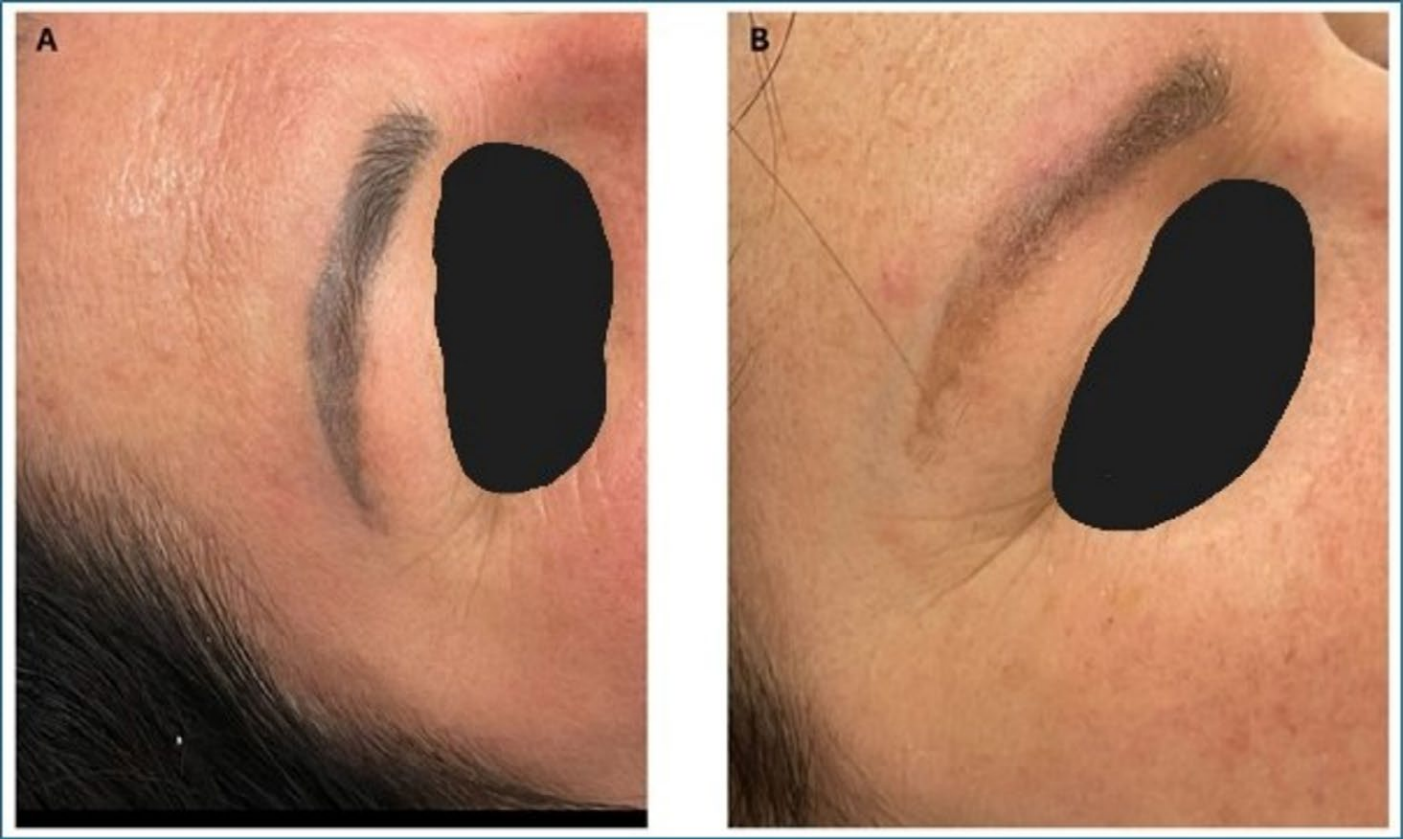
A cosmetic eyebrow tattoo (A) before and (B) after one treatment session.

**FIGURE 3:**
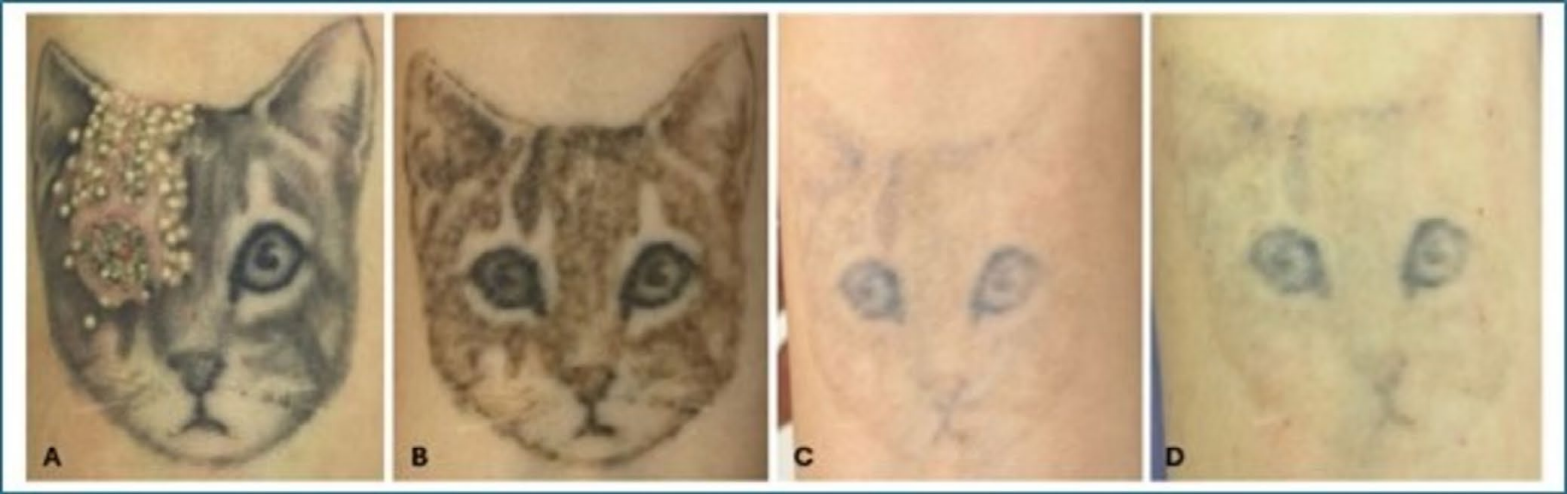
An artistic tattoo (A) During the first treatment session, (B) 1 month following the second session, (C) 1 month following the third session, and (D) 3 months following the last (fourth) treatment session.

**FIGURE 4:**
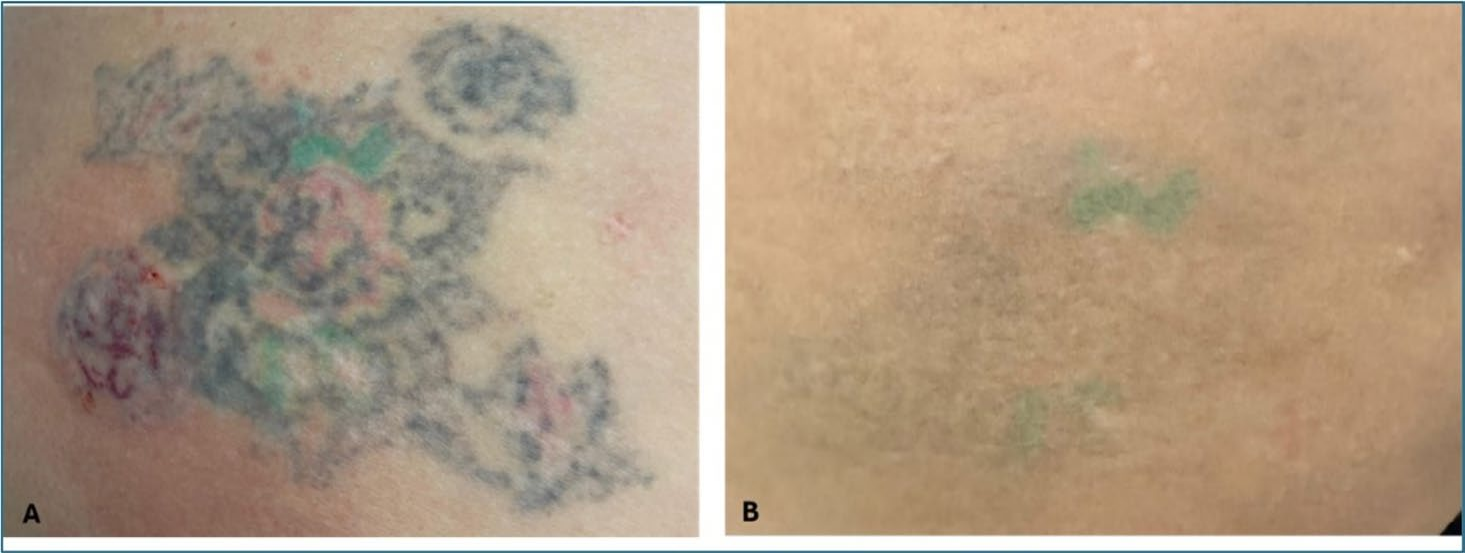
An artistic tattoo that had previously undergone seven treatment sessions at another clinic (A), during the first treatment session (B) 3 months following the third session.

**FIGURE 5:**
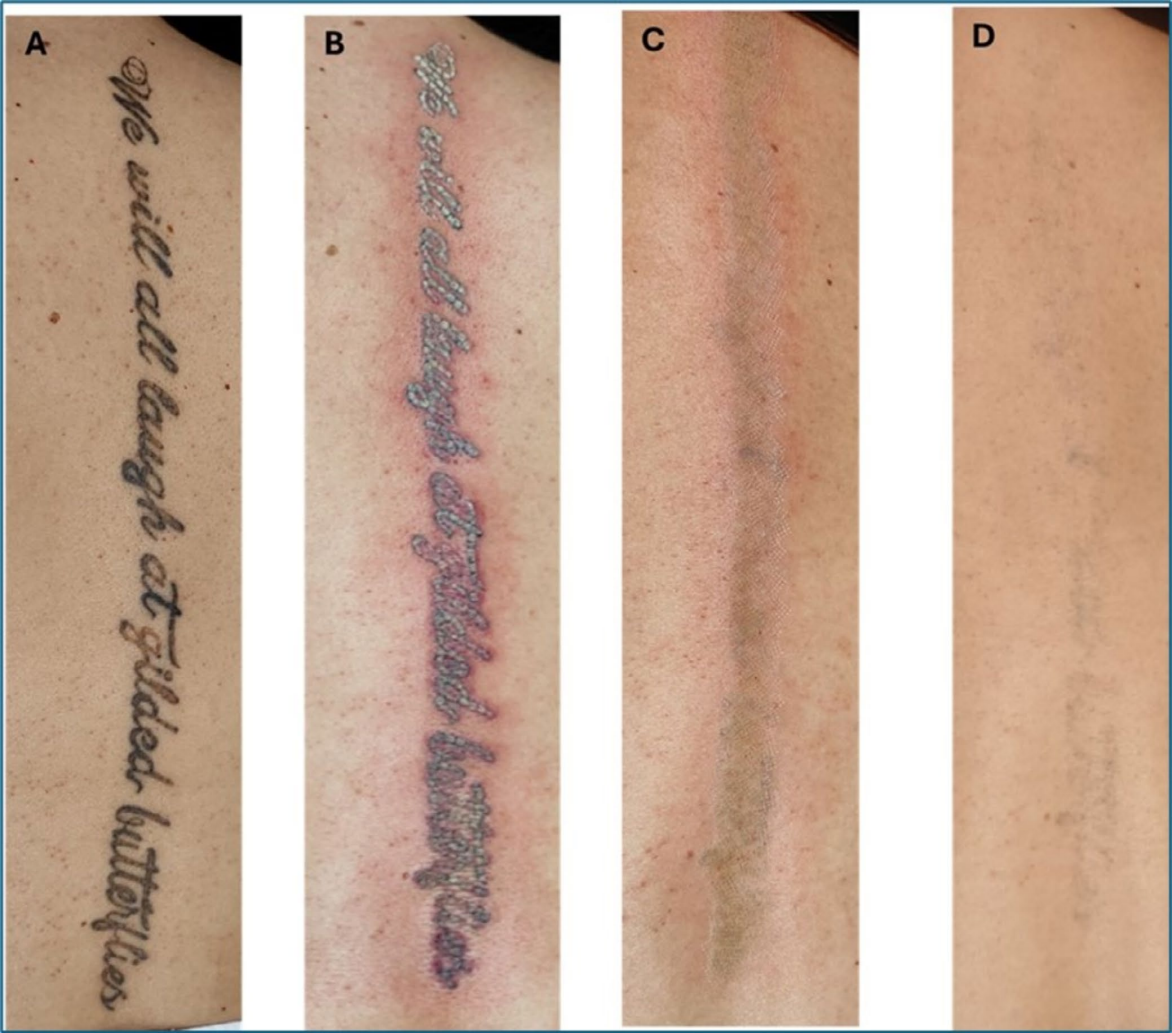
An artistic tattoo along the spine. (A) At baseline, (B) immediately after the first treatment session, (C) 6 weeks following the second treatment, and (D) 12 months following the third treatment.

A total of 111 patients were included in the analysis. The mean age was 33.3 ± 7.8 years, and the majority (88.3%) were females. Most patients had Fitzpatrick skin types II (27.9%) and III (66.7%). The number of treatment sessions ranged from 1 to 8, with a mean of 2.5 ± 1.55. Adjunctive fractional CO_2_ rescue treatment was used in 40 patients (44%) because of insufficient response after three Nd:YAG/KTP treatment sessions. Most sessions were spaced at 6‐ to 7‐week intervals. Approximately one‐third of patients underwent only a single treatment session, and the mean percentage of tattoo clearance was 64.1% ± 18.1%. Roughly half (55%) of the tattoos were monochromatic, and 45% were multicolored. Tattoos were most frequently located on the limbs (45%), followed by the head/neck (29.7%) and the torso (25.2%). A summary of patient and tattoo characteristics is provided in Table 1, treatment characteristics are presented in Table 2, and representative digital images of tattoos are shown in Figures 2, 3, 4, 5.

**TABLE 1:** Patients and tattoos characteristics.

	Level	Overall
*n*		111
Age (years) (mean [SD])		33.33 (7.76)
Gender (%)	F	98 (88.3)
	M	13 (11.7)
Skin type (%)	II	31 (27.9)
	III	74 (66.7)
	IV	6 (5.4)
Body region (%)	Head/neck	33 (29.7)
	Limbs	50 (45.0)
	Torso	28 (25.2)
Tattoo age (years) (mean [SD])		6.28 (4.59)
Tattoo size (%)	Small	48 (43.2)
	Medium	32 (28.8)
	Large	16 (14.4)
	XL	15 (13.5)
Tattoo color (%)	Mono	61 (55.0)
	Multi	50 (45.0)

**TABLE 2:** Treatment characteristics.

	Overall
*n*	111
Adjunctive fractional CO_2_ rescue treatment, *n* (%)	40 (44)
Sessions (mean [SD])	2.51 (1.55)
Intervals (weeks) (%)	
4	9 (8.1)
5	6 (5.4)
6	40 (36.0)
7	24 (21.6)
Single session	32 (28.8)
Hypopigmentation (%)	1 (0.9)
PIH (%)	11 (9.9)
Prolonged erythema (%)	2 (1.8)
Scar (%)	1 (0.9)
None (%)	98 (88.3)
% Clearance (mean [SD])	64.14 (18.10)

### 3.1 Efficacy Outcomes

**FIGURE 6:**
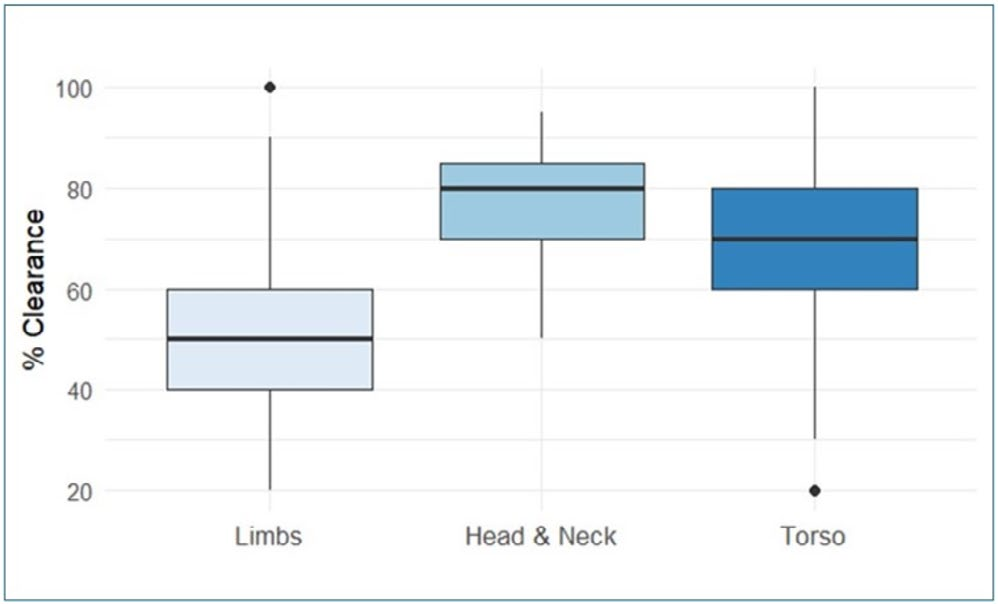
Clearance (%) per tattoo body area.

Patients achieved a mean 64% reduction in visible tattoo pigmentation, with improvement in clearance observed up to 1 year following the final treatment session (Figure 5). Agreement between the two blinded photographic assessors was excellent, with an inter‐rater reliability coefficient of ICC = 0.92 (95% CI, 0.88–0.95). In univariable linear regression models, several factors were significantly associated with the percentage of tattoo clearance. Male gender was associated with a significantly lower clearance than female gender (*β* = −12.10, *p* < 0.05). However, this finding should be interpreted with caution because the male subgroup was small, comprising only 13 patients, whereas 98 were female. Tattoos with multiple colors showed significantly higher clearance compared to monochromatic tattoos (*β* = +10.11, *p* < 0.01). As shown in Figure 6, tattoos on the limbs had significantly lower clearance than those on the head/neck (*β* = −23.32, *p* < 0.001), whereas tattoos on the torso showed a non‐significant reduction (*β* = −7.41). Regarding treatment intervals, patients with 6‐ or 7‐week intervals had significantly lower clearance than other categories (*β* range: −13.47 to −19.76, *p* < 0.05).

In a multivariable linear regression model including all patients (*n* = 111), predictor variables included gender, color category, and body region, which were significant in univariable models. The model was also adjusted for age, skin type, and tattoo size to account for potential confounding. In this model, tattoos on the torso (+16.3%, *p* < 0.001) and head/neck (+16.1%, *p* = 0.002) were associated with significantly greater clearance than tattoos on the limbs, which served as the reference category. Pairwise comparisons of estimated marginal means further supported these findings: clearance rates were significantly lower for tattoos on the limbs compared to those on the head/neck (*p* = 0.0044) and torso (*p* = 0.0002), with no significant difference observed between the head/neck and torso regions (*p* = 0.9995).

**FIGURE 7:**
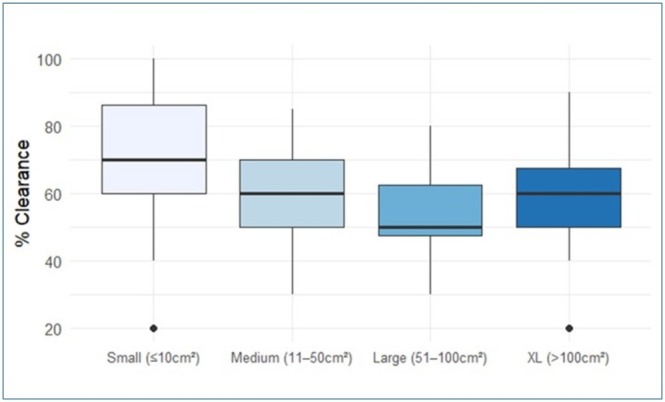
Clearance (%) per tattoo size.

Additionally, as seen in Figure 7, small tattoos (reference) were associated with significantly greater clearance than large tattoos (+12.3%, *p* = 0.033) in the regression model. Medium (−9.1%, *p* = 0.052) and XL tattoos (−6.2%, *p* = 0.29) also showed trends toward lower clearance compared to small tattoos, although these differences were not statistically significant. However, pairwise comparisons of estimated marginal means, adjusted for multiple comparisons, did not reveal statistically significant differences among the size categories. This model explained 33% of the variance (Adjusted *R* ^2^ = 0.334). In the sensitivity analysis excluding patients who received adjunctive fractional CO_2_ rescue treatment, the main body‐region finding remained directionally consistent. Compared with limb tattoos, torso tattoos showed greater adjusted clearance (+16.3%, *p* < 0.001), and head/neck tattoos showed no statistically significant difference in adjusted clearance (*β* = +16.1%, *p* = 0.002). The sensitivity model explained 33.4% of the variance. These findings support the interpretation that anatomical location, particularly the lower clearance observed for limb tattoos, was not solely attributable to the inclusion of fractional CO_2_ rescue cases.

To account for the potential effect of the interval between sessions, a second model was constructed using the subset of patients who underwent more than one treatment session (*n* = 79), with the interval included as an additional predictor. In this group, only the torso location was a statistically significant predictor of clearance compared with the limbs (*p* = 0.001), which served as the reference category. No other predictors reached statistical significance. Pairwise comparisons of estimated marginal means supported these findings: clearance was significantly higher for torso tattoos than for limb tattoos (*p* = 0.0011). However, no significant differences were observed between the head/neck and either the limbs (*p* = 0.314) or the torso (*p* = 0.876). The model explained approximately 26% of the variance in clearance outcomes (Adjusted *R* ^2^ = 0.256).

### 3.2 Safety Outcomes

**FIGURE 8:**
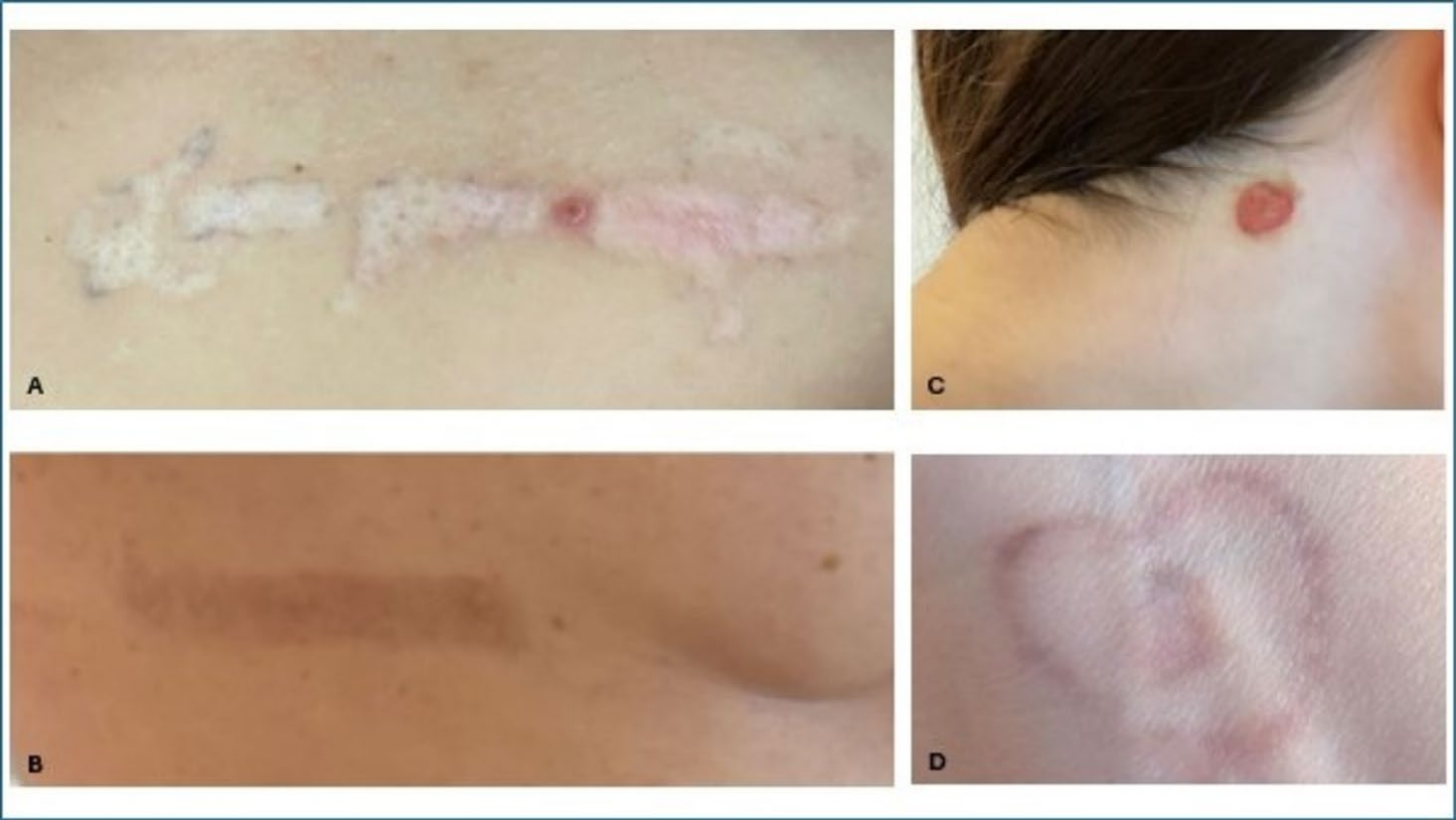
Anticipated and Transient Tattoo Removal Side Effects. Representative digital Images of (A) Hypopigmentation, (B) PIH, (C) Scar, and (D) Prolonged erythema.

**FIGURE 9:**
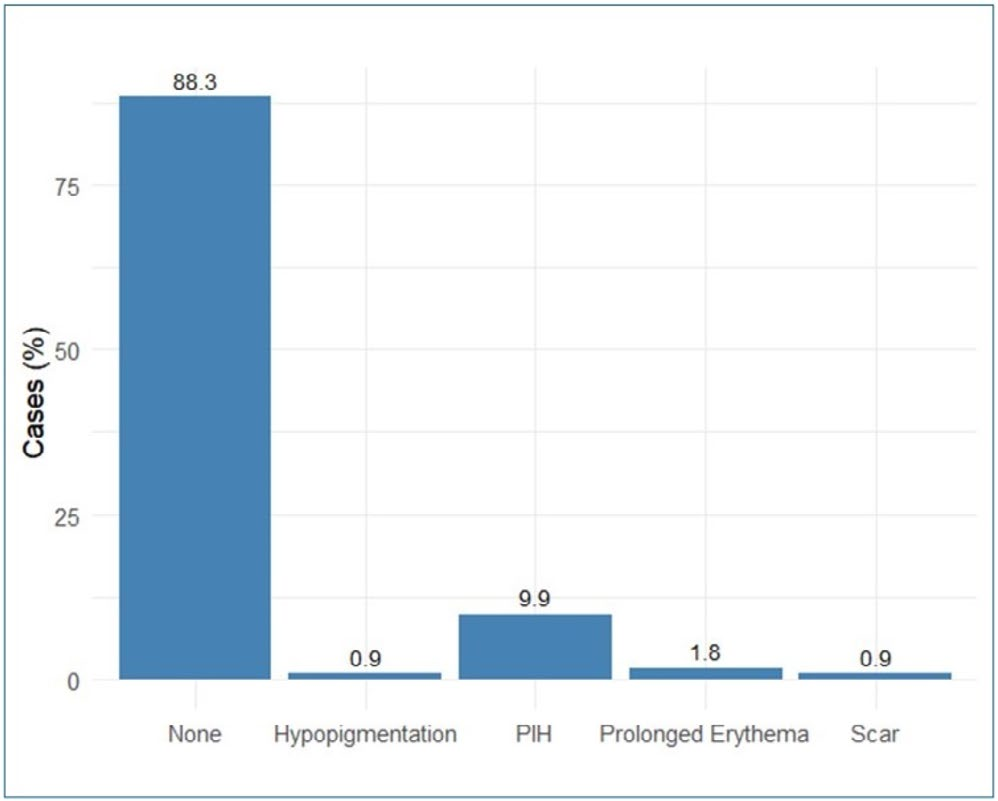
Side effects distribution. Percentages sum to > 100% as 2 patients had both PIH and prolonged erythema.

The treatment demonstrated an excellent safety profile, with the vast majority of patients (88%) experiencing no adverse effects. Some cases had transient and expected post‐inflammatory hyperpigmentation, hypopigmentation, prolonged erythema, and scarring (Table 2; Figures 8 and 9).

A one‐way ANOVA showed no significant association between reported side effects and percentage tattoo clearance, *F*(4, 106) = 1.30, *p* = 0.27.

## 4 Discussion

Although there were many interesting observations in this multi‐center study, the most clinically applicable finding was that anatomical location was the strongest and most consistent predictor of photographic clearance, with tattoos on the trunk and head/neck having statistically higher clearance rates than those on the limbs. The size of the tattoo showed a weaker correlation with photographic clearance and was not consistently significant across all models. The mean clearance rate was 64.1%, and adverse events were rare in this population. Because this study included previously treated tattoos and resistant tattoos treated with adjunctive fractional CO_2_ rescue therapy, the results should be interpreted as real‐world evidence for a Q‐switched Nd:YAG‐centered treatment protocol rather than as evidence for Nd:YAG monotherapy alone. To address this potential confounder, we reported the proportion of fractional CO_2_ rescue cases and performed a sensitivity analysis excluding these patients. The overall clearance and safety results reported by the authors of this paper are consistent with previously published articles, all of which report high clearance, high patient satisfaction rates, and relatively low rates of adverse events following Q‐switched 1064/532 nm Nd:YAG laser therapy. Cannarozzo et al. [10] report a clearance rate of 60% after an average of 4.6 sessions in a double‐center retrospective study, Egozi and Toledano [11] report that Q‐switched Nd:YAG therapy is a safe and effective treatment modality for tattoo removal in a retrospective clinical series, with continued improvement observed even after the final treatment session. Other favorable outcomes have also been reported regarding cosmetic tattoo removal using the same wavelength combination [12]. This study, however, adds to the previously reported body of evidence by focusing more on optimizing treatment effectiveness rather than merely reporting success rates.

The observation that tattoos on the limbs are generally associated with lower clearance rates than those on the trunk and head/neck region is consistent with prior literature on tattoo removal. For example, Bencini et al. [8] found that tattoos on the feet and legs were associated with poor outcomes among the largest cohort ever treated with Q‐switched lasers. It has also been reported in previous papers that several tattoo and patient‐level characteristics may influence clearance rates [7, 8, 9]. Although there are several potential explanations for why limb tattoos would require more sessions than tattoos elsewhere, these include the fact that the area is often harder to reach, has different vasculature, lymphatics, skin characteristics, and sun exposure rates. Alternatively, the difference in clearance may be due to tattoos on the limbs being different from those on the trunk and face. Anatomical location thus acts as both a predictor and a proxy variable.

Smaller‐sized tattoos exhibited a trend toward higher clearance rates; however, this trend was attenuated after controlling for covariates. Other patient variables, including sex and tattoo color category, were statistically significant in univariable analyses but were attenuated after adjustment for other covariates. The apparent association between male sex and lower clearance should be interpreted with particular caution because the cohort was strongly imbalanced by sex, with only 13 male patients compared with 98 female patients. Therefore, the univariable sex effect may reflect sparse subgroup data, differences in tattoo characteristics between men and women, or residual confounding rather than a true biological difference in laser response. This suggests that the anatomical location was perhaps the most significant predictor of treatment response in this population. Additionally, because this study included previously treated and resistant tattoos that underwent fractional CO_2_ laser treatment, the observed outcomes cannot be attributed solely to Nd:YAG monotherapy.

It seems that the safety profile in this study was excellent, since most patients had no complications, and the few patients who did develop an adverse event were either experiencing prolonged erythema or pigmentary alterations. Given that Q‐switched Nd:YAG lasers are considered a safe way to remove tattoos without causing damage to adjacent skin structures, the relatively low adverse event burden in this population is reassuring [13, 14, 15, 16].

This point is particularly relevant because the present cohort included a substantial proportion of patients with Fitzpatrick skin types III–IV. In a recent multicenter study, Cannarozzo et al. [17] evaluated Q‐switched 1064/532‐nm Nd:YAG laser treatment for hyperpigmentation in Asian patients, a population in which higher Fitzpatrick phototypes and post‐laser pigmentary risk are clinically important considerations. The relatively low frequency of pigmentary complications in the present cohort, including PIH in 11 patients and hypopigmentation in 1 patient, is therefore consistent with the broader view that careful parameter selection and appropriate post‐treatment care are central to minimizing pigmentary adverse events in patients with greater baseline melanin content [17].

This study has many strong points: first, it reflects real‐world practices, includes both tattoo types, and does not exclude previously treated or resistant patients. On the other hand, these strengths are the source of many important weaknesses, including the non‐prospective design of this study, the multicenter nature, which increases heterogeneity, the absence of a standardized follow‐up, the absence of a blinded photographer who estimated tattoo clearance, and finally, the lack of important information on individual patient characteristics. These weaknesses affect the authors' conclusions regarding the statistical significance of the observed associations and reduce our ability to draw causal inferences from this study. Although a sensitivity analysis excluding fractional CO_2_ rescue cases was performed, residual confounding cannot be excluded because the decision to add fractional CO_2_ treatment was based on insufficient response after prior sessions and, therefore, identified a clinically resistant subgroup.

## 5 Conclusions

With the increasing popularity of tattoo removal treatments, it is important that we have scientific data to establish reasonable treatment expectations for patients. Based on this multicenter retrospective cohort study, one can expect high rates of photographic clearance in both artistic and cosmetic tattoos using a Q‐switched 1064/532 nm Nd:YAG protocol, with infrequent adverse events in a patient population that includes both previously treated and resistant tattoos treated with fractional CO_2_ laser. The strongest predictor of low response is the presence of tattoos on the limbs.

## Author Contributions

Shaked Menashe: supervision, project administration, and writing – review and editing. Sharon Moscovici: data curation, formal analysis, and investigation. Omer Dor: data curation, investigation. Yoad Govrin: data curation, investigation. Gerta Kapxhiu: data curation, formal analysis, and investigation. Arminda Avdulaj: conceptualization, methodology, and writing of original draft.

## Funding

The authors have nothing to report.

## Ethics Statement

This study was conducted in accordance with the Declaration of Helsinki. Ethical approval was obtained from the Institutional Ethics Committee of Shamir Medical Center on October 9th, 2024 (approval number: ASF‐0202‐24).

## Consent

The patients in this manuscript have given written informed consent to publication of their case details and any accompanying images. A copy of the written consent is available for review by the Editor‐in‐Chief of this journal.

## Conflicts of Interest

The authors declare no conflicts of interest.

## Data Availability

The data supporting the findings of this study are available from the corresponding author upon reasonable request.
